# Programmed death-ligand 1 (PD-L1) expression in various tumor types

**DOI:** 10.1186/2051-1426-1-S1-P53

**Published:** 2013-11-07

**Authors:** Joseph Grosso, David Inzunza, Qiuyan Wu, Jason Simon, Parul Singh, Xiaoling Zhang, Therese Phillips, Pauline Simmons, John Cogswell

**Affiliations:** 1 Bristol-Myers Squibb, Princeton, NJ, USA; 2 Dako North America, Inc., Carpinteria, CA, USA

## Background

Programmed cell death-1 (PD-1) is a co-inhibitory receptor expressed on lymphoid and non-lymphoid-derived cells that negatively regulates peripheral T-cell responses. PD-L1, the major PD-1 ligand, is expressed on various tumors and is being investigated as a possible predictive marker for anti-PD-1 therapy. Few studies have determined PD-L1 expression across tumor types using a consistent process. We evaluated PD-L1 expression across human tumor samples using a novel, automated, sensitive, and specific PD-L1 IHC assay (developed by Dako) using the 28-8 antibody.

## Methods

PD-L1 expression in 654 commercially available tumor samples was explored. Percentage of tumor plasma membrane staining was determined at ≥1+ intensity. Macrophage and lymphocyte PD-L1 status was determined by compartment (tumor, non-tumor associated, or both). Immune cell density and PD-L1+ immune cell frequency were assessed on a scale of 0 (none), 1 (mild), 2 (moderate), and 3 (heavy). PD-L1 positivity was evaluated using 5% surface expression values for tumor cell membrane staining.

## Results

Of 654 samples examined, spanning 19 tumors from different sites, 89 (14%) were PD-L1+ (≥5% frequency). Highest PD-L1+ frequencies were seen in head and neck (17/54; 31%), cervical (10/34; 29%), cancer of unknown primary origin (CUP; 8/29; 28%), glioblastoma multiforme (GBM; 5/20; 25%), bladder (8/37; 21%), esophageal (16/80; 20%), triple negative (TN) breast (6/33; 18%), and hepatocarcinoma (6/41; 15%). Across a subset of head and neck tumor samples, high PD-L1 expression was seen: lip (2/2; 100%), tongue (2/4; 50%), larynx (11/31; 35%), and oral cavity (2/10; 20%). In breast tumors, TN tumors showed a higher frequency PD-L1+ status (≥5% frequency; 6/33 samples [18%]) compared with estrogen receptor, progesterone receptor, or HER2+ positive tumors (0/45). PD-L1+ was higher in squamous (SQ) compared with other histologies within esophageal (SQ [14/55; 25%] vs other [2/25; 8%]), cervical (SQ [6/15; 40%] vs adenoSQ [2/8; 25%] and adeno [0/5]), and bladder (SQ [3/8; 37%] vs transitional [6/27; 22%]) tumors. PD-L1+ tumors were associated with immune cell density and PD-L1+ immune cells, especially in TN breast and laryngeal tumors.

## Conclusions

PD-L1 is expressed in various human tumors and is associated with tumor grade, squamous histology, immune cell density, and co-localization of PD-L1+ immune cells. Highest PD-L1+ frequencies were seen in head and neck, cervical, CUP, GBM, bladder, esophageal, TN breast cancer, and hepatocarcinoma.

